# The Serum ACE2, CTSL, AngII, and TNFα Levels after COVID-19 and mRNA Vaccines: The Molecular Basis

**DOI:** 10.3390/biomedicines11123160

**Published:** 2023-11-27

**Authors:** Mina Pencheva, Martina Bozhkova, Yordan Kalchev, Steliyan Petrov, Alexandra Baldzhieva, Teodora Kalfova, Valentin Dichev, Donka Keskinova, Silvia Genova, Mariya Atanasova, Mariana Murdzheva

**Affiliations:** 1 Department of Medical Physics and Biophysics, Faculty of Pharmacy, Medical University of Plovdiv, 4002 Plovdiv, Bulgaria; 2 Department of Medical Microbiology and Immunology, Faculty of Pharmacy, Medical University of Plovdiv, 4002 Plovdiv, Bulgaria; martina.bozhkova@mu-plovdiv.bg (M.B.); yordan.kalchev@mu-plovdiv.bg (Y.K.); steliyan.petrov@mu-plovdiv.bg (S.P.); alexandra.baldzhieva@mu-plovdiv.bg (A.B.); teodora.kalfova@mu-plovdiv.bg (T.K.); mariya.atanasova@mu-plovdiv.bg (M.A.); mariana.murdzheva@mu-plovdiv.bg (M.M.); 3 Research Institute, Medical University of Plovdiv, 4002 Plovdiv, Bulgaria; valentin.dichev@mu-plovdiv.bg; 4 Department of Medical Biology, Medical University of Plovdiv, 4002 Plovdiv, Bulgaria; 5 Department of Applied and Institutional Sociology, Faculty of Philosophy and History, University of Plovdiv “Paisii Hilendarski”, 4000 Plovdiv, Bulgaria; d.keskinova@uni-plovdiv.bg; 6 Department of General and Clinical Pathology, Medical Faculty, Medical University of Plovdiv, 4002 Plovdiv, Bulgaria; sylvia_genova@abv.bg; 7 Laboratory of Virology, UMBAL “St. George” EAD, 4002 Plovdiv, Bulgaria

**Keywords:** biomarkers, COVID-19, cytokines, inflammation, immune response, mRNA vaccines

## Abstract

Background: The SARS-CoV-2 virus as well as the COVID-19 mRNA vaccines cause an increased production of proinflammatory cytokines. Aim: We investigated the relationship between ACE2, CTSL, AngII, TNFα and the serum levels of IL-6, IL-10, IL-33, IL-28A, CD40L, total IgM, IgG, IgA and absolute count of T- and B-lymphocytes in COVID-19 patients, vaccinees and healthy individuals. Methods: We measured the serum levels ACE2, AngII, CTSL, TNFα and humoral biomarkers (CD40L, IL-28A, IL-10, IL-33) by the ELISA method. Immunophenotyping of lymphocyte subpopulations was performed by flow cytometry. Total serum immunoglobulins were analyzed by the turbidimetry method. Results: The results established an increase in the total serum levels for ACE2, CTSL, AngII and TNFα by severely ill patients and vaccinated persons. The correlation analysis described a positive relationship between ACE2 and proinflammatory cytokines IL-33 (r = 0.539) and CD40L (r = 0.520), a positive relationship between AngII and CD40L (r = 0.504), as well as between AngII and IL-33 (r = 0.416), and a positive relationship between CTSL, total IgA (r = 0.437) and IL-28A (r = 0.592). Correlation analysis confirmed only two of the positive relationships between TNFα and IL-28A (r = 0.491) and CD40L (r = 0.458). Conclusions: In summary, the findings presented in this study unveil a complex web of interactions within the immune system in response to SARS-CoV-2 infection and vaccination.

## 1. Introduction

COVID-19 surprised the world with its scale of injury impact, complexity of pathological manifestations and the number of patients who lost the battle. Since December 2019, SARS-CoV-2 has become a world crisis for the public health, which has led to high mortality rates worldwide [1,2]. COVID-19 is characterized by infections to the upper and lower respiratory tract, leading to asymptomatic or diverse clinical symptoms, including cough, fever and pneumonia, accompanied by other complications such as diarrhea and multiorgan failure [3,4,5].

The disease is characterized by an initial viremic phase followed by a hyperinflammatory phase, which, in some patients, transforms into a self-reinforcing dysregulated immune response, ending in high mortality [6]. In this complex situation, the elderly were the most affected, in whom COVID-19 caused higher morbidity and mortality, as well as a higher risk of complications due to the presence of other co-morbidities [7]. 

Although most people recovered successfully from the infection, the long-term consequences from the viral interaction are still the main topic of research for many specialists. In order to reduce the consequences of both COVID-19 and long COVID-19, it is necessary to prevent infection by vaccination, on the other hand, to continue conducting research to track the factors and mechanisms that underlie COVID-19 [8].

In the beginning of the pandemic, it was shown that the glycoprotein of SARS-CoV-2 interacts with the angiotensin converting enzyme 2 (ACE2), ensuring viral entry in the host cells [9]. The main route of penetration inside the cell is by internalization, after connection to ACE2, followed by Spike protein cleavage, which gives rise to two subunits—S1 and S2. This is mediated by transmembrane serine protease 2 (TMPRSS2) [9], facilitating viral penetration. Due to the importance of both ACE2 and CTSL in the context of COVID-19 [10,11], it is important for those biomarkers to be studied in order to evaluate whether they have an impact on the immune response and disease severity. 

After viral attachment and the internalization of ACE2, the balance in the ACE/AngII/AT1R and ACE2/Ang 1-7/MasR axes becomes impaired [6]. Angiotensin II (AngII) can activate the nuclear factor kappa B (NF-κB) pathway [12] via stimulation of the phosphorylation of the p65 subunit [13]. This leads to an increased production of IL-6, TNFα, IL-1B and IL-10 [14]. Tumor necrosis factor α (TNFα) is the main engine for the activation of the NF-κB signaling pathway, inducing the expression of both proinflammatory and antiapoptotic genes [15,16]. Monitoring ANGII levels can help assess the influence of the renin-angiotensin system (RAS), in which the ANGII is involved, on immune responses and disease outcomes, particularly in severe cases.

On the other hand, an activation of innate lymphoid cells occurs after the initial SARS-CoV-2 infection. These cells release proinflammatory cytokines, such as IL-1, IL-6, IL-8, IL-12, TNFα and some chemokines [17]. The release of inflammatory molecules recruits additional lymphoid cells (monocytes, macrophages, neutrophiles, dendritic cell (DC), and natural killer cells (NK cells)) and activate cells, belonging to the adaptive immunity (CD4+ and CD8+ T lymphocytes) from nearby tissues, which can secrete IL-2, IFN-γ and TNFα, leading to an acceleration of the damage [18]. These humoral biomarkers represent a comprehensive panel of immune factors that are critical in shaping the immune response to SARS-CoV-2. In addition, CD40L and IL-28A are associated with B cell activation and antibody production, crucial components of the adaptive immune response. Cytokines such as IL-10 and IL-33 are involved in modulating the immune response and inflammation. Studying these humoral biomarkers can shed light on the interplay between the adaptive and innate immune responses, autoimmune phenomena and immune regulation in the context of COVID-19 and vaccination. 

The result of an uncontrolled lung inflammation causes a cytokine storm, followed by acute respiratory distress syndrome (ARDS), which can be observed in patients with severe COVID-19 [19].

In order to prevent the spread of the virus among people, limit the pandemic and reduce the incidence of severe forms of COVID-19 (reducing both the morbidity of COVID-19 and the mortality of persons infected with SARS-CoV-2), the World Health Organization (WHO) has approved the safety and efficacy of several vaccines against COVID-19, including the products of Oxford–AstraZeneca, Johnson and Johnson, Pfizer-BioNTech and Moderna, etc. [20]. 

Identification and quantification of biomarkers 2 weeks after complete vaccination and 14 days after RT-PCR confirmed SARS-CoV-2 infection is important to provide information on both post-infection and vaccine-induced protective immune responses, disease severity and host–pathogen interactions.

The purpose of this study is to determine the levels of expression of ACE2, CTSL, AngII and TNF-α, which are critical participants in the process of virus invasion and the immune response from the host. Conversely, SARS-CoV-2 impairs the signaling pathways associated with these biomarkers, which are essential in the pathogenesis of COVID-19 across multiple stages of the disease, including COVID-19 pneumonia and vaccination. 

## 2. Materials and Methods

### 2.1. Study Design

In this prospective study, we included a total of 149 people, divided into 3 groups: (1) hospitalized COVID-19 patients with moderate and severe course of the infection (*n* = 63), median age 72 (23–87); (2) vaccinees (*n* = 49), median age 45 (27–60); (3) healthy individuals (HI) with no clinical and immunological signs of COVID-19 (*n* = 37), median age 54 (35–69). The laboratory results and data for experimental variables of the COVID-19 patients are available in Supplementary Table S1.

We assessed the expression of serum levels of ACE2, CTSL, AngII and TNF-α in patients with COVID-19 (on day 14 after SARS-CoV-2 PCR confirmation), vaccinees (14 days after the second dose of mRNA vaccine) and healthy individuals.

### 2.2. Participants and Sample Collection

Patients were recruited during hospitalization at the Clinic of Infectious Diseases, University Multiprofile Hospital for Active Treatment “St. George”-Plovdiv, while vaccinated and healthy individuals were selected from the Medical University of Plovdiv, Bulgaria, between October 2021 and April 2022.

Hospitalized patients with COVID-19 were confirmed to have SARS-CoV-2 infection using real-time RT-PCR (GeneLEAD VIII apparatus and COVID-19 Real Time Multiplex RT-PCR Kit, Labsystems). Form of the disease was classified according to WHO: severe patients exhibiting oxygen saturation <90% on room air at sea level, signs of pneumonia and ARDS.

Vaccinated individuals received two doses of either Pfizer-BNT162b2 (Pfizer Inc. New York, NY, USA) or mRNA-1273 Moderna vaccines (Moderna Inc. Cambridge, MA, USA). The control group was tested negative for COVID-19 using an antigen test (Aurora Biomed Inc., Vancouver, BC, Canada) and had no clinical signs of active infection.

All subjects in the study were randomly selected and were heterogeneous in terms of age, gender, and clinical presentation.

Venous blood samples were collected from COVID-19 patients and vaccinated individuals on the 14th day after infection registration or after receiving the second vaccine dose, respectively. Blood was collected from healthy control subjects on the day of the antigen test. After venipuncture, the serum was separated within 4 h and stored at −80 °C until further use.

### 2.3. Inclusion/Exclusion Criteria

The main inclusion criteria for this study were as follows: (a) age above 18 years; (b) ability to provide informed consent; (c) individuals admitted and registered with COVID-19 or eligibility for vaccination according to the national COVID-19 vaccination program. 

All individuals in the control group were in good health with no symptoms of upper or lower respiratory tract infections, no history of COVID-19 and not vaccinated against COVID-19. The healthy individuals signed an informed consent and were aged above 18 years.

The exclusion criteria were (a) systemic autoimmune disorders under immunosuppressive therapy; (b) active malignancies; (c) active acute infections; (d) or other immune-mediated disorders.

### 2.4. Methods

#### 2.4.1. Sample Processing and Storage

Blood samples were centrifuged (Shimadzu UV160A, S. No: 28006648, Kyoto, Japan) at 3000 rpm for 10 min, and sera were collected and stored at −80 °C. Prior to the study, the homogenates, which were defrosted at room temperature, were later centrifuged at +4 °C for 5 min at 3000 rpm (Sigma 3K30, S.No: 76262, Sigma Laborzentrifugen GmbH, Osterode am Harz, Germany). The sera supernatants were collected for biochemical analysis. 

PBMCs were isolated from whole peripheral blood via centrifugation in density gradient (Histopaque^®^-1077, Merck KGaA, Darmstadt, Germany). Isolated cells were mixed with freezing media, stored in cryotubes at −80 °C (Nalgene^®^ Mr. Frosty) and later transferred in liquid nitrogen. 

#### 2.4.2. Enzyme Linked Immunosorbent Assay

The concentrations of ACE2, CTSL, AngII and TNFα in the serum were measured using commercially available SunRed Human ACE2 ELISA kits (SunRed Biological Technology Co., Ltd., Shanghai, China). The sensitivity of the ACE2 kit was 9825 pg/mL, CTSL kit was 37.5 pg/mL, AngII kit was 3125 pg/mL and the range of the assay was 62.5–4000 pg/mL. The sensitivity of TNFα kit was 4.32 pg/mL, and the range of the assay was 15.63–1000 pg/mL. 

Due to the lack of reference values to account for an increase or decrease in the expression levels of the markers included in this study, we adopted the average value in healthy patients as a reference according to which we could determine an increase or decrease in the values of the investigated markers. Based on the values of the healthy persons, we divided the patients with COVID-19 into patients with moderate COVID-19 and severe COVID-19, according to each of the 4 biomarkers.

Assessment of humoral biomarkers (CD40L, IL-28A, IL-10, IL-33) was performed via ELISA (SunRed BiologicaL Technology Co., Shanghai, China), and absorption was measured at 450 nm on BioTek^®^800™TS bioreader (BioTek Instruments, Inc., Winooski, VT, USA). 

#### 2.4.3. Flow Cytometry

Immunophenotyping of lymphocyte subpopulations in whole peripheral blood was performed by flow cytometry (FACSCantoII) using standardized methodology, for which BD Multitest™ 6-color TBNK reagent with TruCount tubes (#662995, BD biosciences, San Jose, CA, USA) was used, containing the following fluorochrome-conjugated antibodies: CD3-FITC/CD16 PE + CD56-PE/CD45-PerCP-Cy™5.5/CD4-PE-Cy™7/CD19-APC/CD8-APC-Cy™7.

#### 2.4.4. Turbidimetry

Total serum immunoglobulins (IgA, IgG, IgM) were analyzed by turbidimetry using a SPAPlus automated analyzer (The Binding Site, Birmingham, UK) and reported as gram per liter (g/L). 

#### 2.4.5. Enzyme-Linked Fluorescent Assay

Anti-SARS-CoV-2 IgG and IgM serum levels were assessed via an enzyme-linked fluorescent assay (ELFA) on VIDAS PC (Biomerieux, SA, Hazelwood, MO, USA) and reported as binding antibody units per milliliter (BAU/mL) after multiplying the antibody index by 20.33.

#### 2.4.6. Data Analysis

Quantitative data were analyzed using the GraphPad Prism software (GraphPad Software 8.0.1 version, Inc., La Jolla, CA, USA). Results are presented with the mean and standard deviation (mean ± SD). Mean values for biomarker levels in the three groups were compared with the Games-Howell test, post-hoc tests for unequal variances to ANOVA. Serum markers in the moderate and severe COVID-19 groups were compared using the Mann–Whitney test. Correlation between the analyzed biomarkers was assessed with Spearman’s rho Correlation Coefficient. Statistical analysis was performed on IBM SPSS Statistics (v.25), where statistical significance was considered at *p* < 0.05, *p* < 0.0001.

## 3. Results

In our study, we reported a change in the cytokine profile, total serum antibody (IgM, IgG) titers and viral-specific antibodies in the group of COVID-19 patients and vaccinated subjects (with two doses of mRNA vaccine) in comparison to the healthy subjects.

Infection with the SARS-CoV-2 virus causes changes in the expression levels of the biomarkers we studied such as ACE2, CTSL, AngII and TNF-α, which, in turn, affect the expression of some cytokines, absolute counts of B and T lymphocytes, as well as the development of specific antibodies against the pathogen. To evaluate the effect of the virus-induced levels of ACE2, CTSL, AngII and TNF-α on different serum markers, we compared two independent excerptions and measured the correlation between the main biomarkers and the serum cytokine levels.

### 3.1. Serum Levels of ACE2, AngII, CTSL and TNFα Induced by COVID-19, BNT162b2 mRNA and mRNA-1273 Vaccines

**Figure 1:**
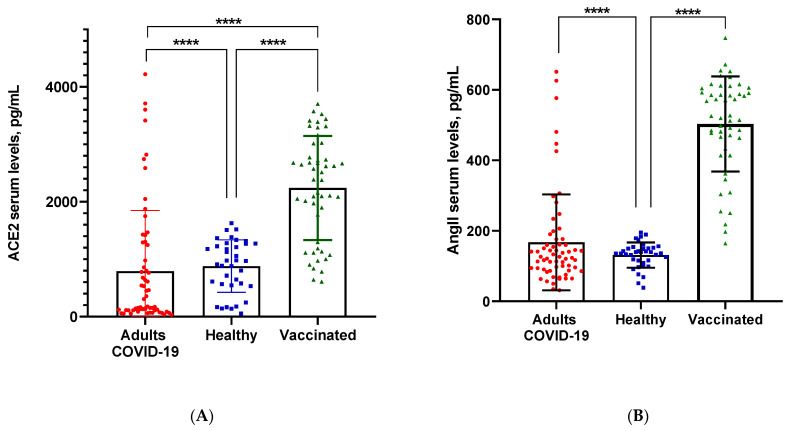

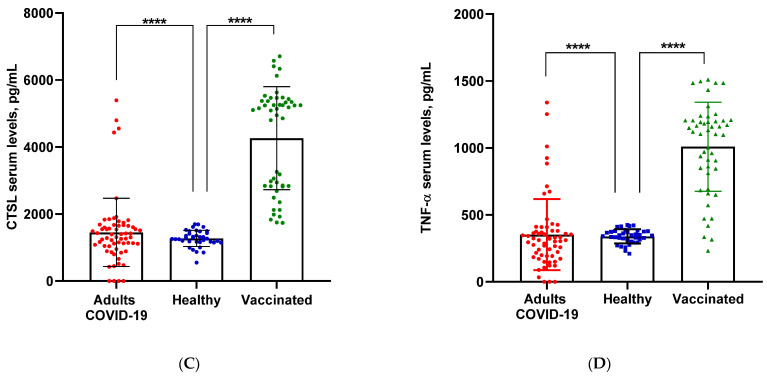
The data illustrate the mean values and standard deviation (mean ± SD) of (**A**) ACE2; (**B**) CTSL; (**C**) AngII; and (**D**) TNF-α in the group of COVID-19 patients, vaccinated individuals and healthy subjects. The comparisons between these groups were carried out using the column dot plot. Asterisk indicates significant differences between groups—**** *p* < 0.0001.

The measured total serum levels for ACE2 in healthy patients were the lowest (325.34 ± 311.78 pg/mL). We reported an increase in patients with severe COVID-19 (1332.21 ± 535.05 pg/mL, *p* < 0.001), as the highest levels of serum ACE2 were observed in vaccinated patients (2240.79 ± 882.53 pg/mL, *p* < 0.001) (Figure 1A). Individual comparisons of values between the serum levels of vaccinated individuals and deceased severe COVID-19-infected individuals were very close.

Statistical analysis found significant values of AngII in the healthy (131.23 ± 25.50 pg/mL) versus vaccinated groups (502.98 ± 107.91 pg/mL, *p* < 0.001). However, we found no statistical difference between healthy and COVID-19 patients (167.27 ± 136.03 pg/mL, *p* < 0.001) (Figure 1B).

Comparing serum levels of CTSL, we again found that the vaccinated persons (4264.26 ± 1523.44 pg/mL) had the highest values compared to all other groups. Mean values in the group of severe COVID-19 (1452.89 ± 1016.49 pg/mL, *p* < 0.001) were slightly higher than those in healthy individuals (1270.61 ± 173.47 pg/mL) (Figure 1C).

Measurement of serum TNFα levels resulted in almost three-fold-higher values in vaccinees (1009.14 ± 296.17 pg/mL) compared to healthy (339.62 ± 37.60 pg/mL) subjects and patients with severe COVID-19 (350.01 ± 264.70 pg/mL, *p* < 0.001) (Figure 1D).

### 3.2. Correlation Analysis between Serum Levels of Key Biomarkers and Cytokines, Induced by COVID-19

**Figure 2:**
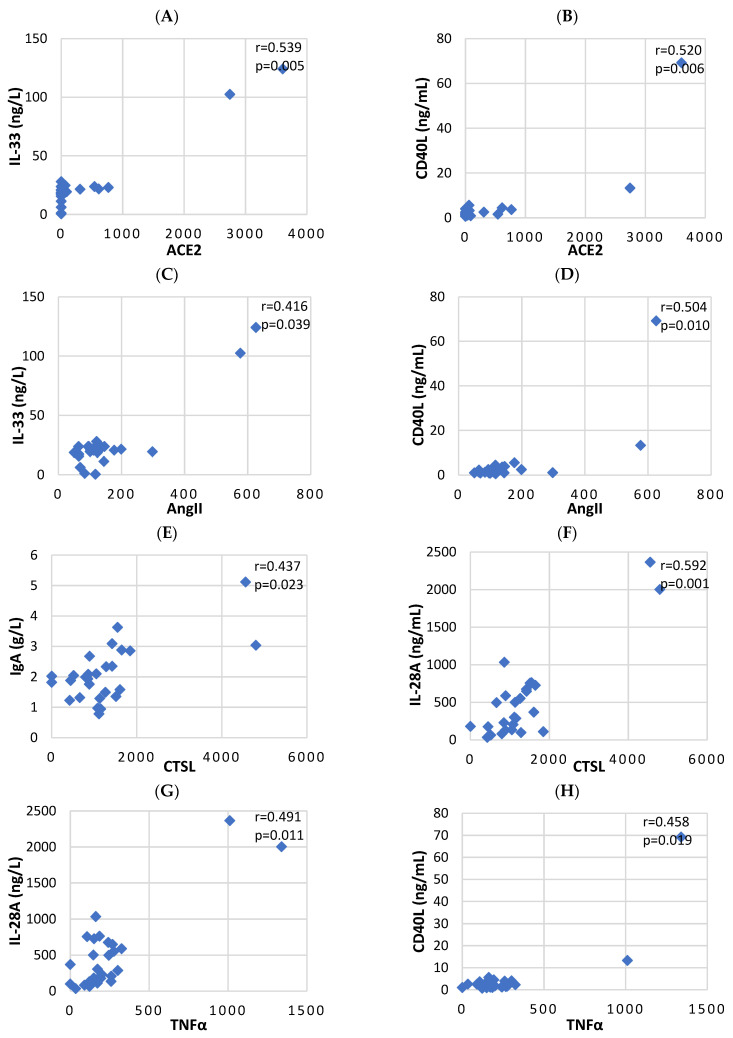
A statistically significant positive link was observed between (**A**) ACE2 and IL-33; (**B**) ACE2 and CD40L; (**C**) AngII and IL-33; (**D**) AngII and CD-40L; (**E**) CTSL and IgA; (**F**) CTSL and L-28A; (**G**) TNF-α and IL-28A; and (**H**) TNF-α and CD-40L. Spearman’s rho correlation coefficient was used to examine all of them.

The values measured on day 14 (after confirmation of the infection) showed a decrease in total serum levels of IgM (*p* = 0.042) and an increase of IL-33 (*p* = 0.009), CD40L levels (*p* = 0.006) and NK cells (*p* = 0.049) in the group of patients with severe COVID-19 versus those with moderate COVID-19 in terms of ACE2 levels (Table 1). The correlation analysis determined the positive relationship between ACE2 and proinflammatory cytokines IL-33 (r = 0.539) and CD40L (r = 0.520) as statistically significant (Table 2; Figure 2A,B).

Analyzing the data regarding the influence of AngII on the expression of the tested markers, we found that in the group of severely ill patients, the values were higher only regarding the proinflammatory cytokine CD40L (*p* = 0.023) (Table 1). Correlation analysis confirmed the positive relationship between AngII and CD40L (r = 0.504) and revealed the relationship between AngII and IL-33 (r = 0.416) (Table 2; Figure 2C,D).

Regarding the influence of CTSL and the comparison of the mean values in the separated groups according to the severity of COVID-19 and the correlation analysis, a positive relationship between CTSL, total IgA (*p* = 0.005, r = 0.437) and IL-28A was established (*p* = 0.013, r = 0.592) (Table 1 and Table 2; Figure 2E,F).

Concerning the influence of TNF-α, we found higher values in the group with severe COVID-19 in terms of IgA, IL-10, IL-33, IL-28A and CD40L and lower values of IgM (Table 1). Correlation analysis confirmed only two of the positive relationships between TNFα, IL-28A (r = 0.491) and CD40L (r = 0.458) (Table 2; Figure 2G,H).

We did not find statistically significant difference between the SARS-CoV-2-specific IgG and IgM levels between patients’ groups (moderate, severe). Although a tendency was observed toward higher virus-specific antibody levels in the severe group for both IgG (mean = 586.32 BAU/mL) and IgM (mean = 206.96 BAU/mL) compared to patients with moderate COVID-19 (IgG mean = 498.69 BAU/mL; IgM mean = 118.32 BAU/mL).

We tried to compare the values of the investigated biomarkers between two groups depending on the SARS-CoV-2 vaccine platform. There were no significant differences in levels of our biomarkers between the two vaccines.

## 4. Discussion

To determine the effect of COVID-19 and vaccines on human health, we examined serum levels of ACE2, CTSL, AngII and TNFα in hospitalized adult patients 14 days after the onset of COVID-19 infection, healthy controls and subjects vaccinated with two doses of COVID-19 mRNA vaccines. 

### 4.1. *Angiotensin Converting Enzyme 2* Serum Levels

We measured the serum levels of ACE2, a major determinant of viral infectivity. After the binding of the virus to ACE2, proteolytic processing of ACE2 follows, which leads to a change in the expression levels of ACE2. All these processes provide information about the progression of the infection and the therapeutic effectiveness. However, it is not fully understood how different serum levels of ACE2 affect the immune response and virulence of SARS-CoV-2.

Our data on ACE2 levels in the serum of patients affected by COVID-19 differ quantitatively in moderate and severe disease, possibly reflecting its changes in tissues at different stages of viral infection. Consistent with previous reports, we found increased levels of anti-ACE2 antibodies in elderly severely ill patients with COVID-19 compared to healthy subjects [21].

According to earlier studies, elevated levels of ACE2 may result from increased shedding of ACE2 due to lysis of the latter from the cells that express it on their surface. This results from the severe lung infection typical for COVID-19 [22].

When analyzing the results from the vaccinees, we discovered higher levels of ACE2 compared to the severe COVID-19 group. We also found a statistically significant increase in CTSL, AngII and TNFα values in vaccinated individuals compared to both healthy controls and COVID-19 patients. Usually, this is expected to be seen in patients due to the fact that the contact mechanism of SARS-CoV-2 with the host cell, as well as the Spike protein synthesized by the cells after administration of mRNA vaccine against COVID-19 [23], use the same receptor, ACE2 [24]. The internalization of ACE2 leads to an imbalance in the RAS system and an increase in AngII activity [25] using TMPRSS2, or lysosomal cathepsin L to cleave the S protein, supporting viral entry in cellular compartment [26,27,28]. In our case, those higher levels observed in the group of vaccinees can be explained by CTSL’s involvement in immune cell activation and antigen processing for further presentation on the surface of MHC molecules. The lower levels in the group of COVID-19 patients may have been due to the dysregulation of the immune response, caused by different factors (age, possible comorbidities, etc.) during the actual infection, which does not occur when one is vaccinated with mRNA vaccine. Furthermore, it is important to take into consideration the viral load in the body. In the hospitalized patients, the viral load could be variable; thus, the immune response might be uneven among all people. As for the viral mRNA composing the vaccine, it is presented in a standardized quantity. AngII and TNFα are expected to be mutually elevated since the former can stimulate the gene expression of the latter. 

In this study, we investigated the interaction between ACE2, CTSL, AngII and TNFα as the main biomolecules actively involved in the process of realization of the immune response after the viral counterplay with the host.

SARS-CoV-2, entering cells, induces the expression of ACE2 and the production of cytokines such as IL-6 (a key immunopathological factor) [29,30], IL-10, IL-28A and CD40L [31,32]. As the severity of the disease increased, we found an increased influence of the investigated markers on cytokine levels, which increased many times. Simultaneously, we reported a decrease in serum IL-6 levels in patients with a severe form of the disease, with the only increase in IL-6 levels observed under the influence of AngII, whose pleiotropic effects we hypothesize are due to these results, also found by other researchers [33]. AngII can stimulate the production of IL-6 in various inflammatory conditions [34]. On the other hand, IL-6 can also contribute to AngII production and its effects. IL-6 can stimulate the synthesis and secretion of AngII in certain cell types, particularly endothelial cells. Additionally, IL-6 can promote vascular dysfunction and oxidative stress, which are factors that can contribute to the generation of AngII and exacerbate its effects on blood pressure regulation and inflammation [35]. The interaction between IL-6 and AngII can create a positive feedback loop that amplifies inflammation and exacerbates the pathophysiology of certain diseases, including cardiovascular diseases, where both IL-6 and AngII play significant roles. 

On the other hand, Sang et al. [36] found that the ACE2 and IL-6 genes have significantly higher positive epigenetic markers for modifications in COVID-19 susceptible individuals compared to non-susceptible. These findings characterize the ACE2 and IL-6 genes as non-canonical interferon-stimulated genes (non-ISGs) that respond differently to inflammatory and interferons (IFNs) signaling than canonical ISGs [36].

Similar results were reported by Maza et al. [37] who found that sera from seronegative individuals could neutralize SARS-CoV-2 in in vitro cellular assays more strongly than sera from unexposed negative controls, despite lacking anti-SARS-CoV-2 IgG antibodies. These results suggest that high serum ACE2 levels may somewhat protect against active infection without generating an increase in antibody levels.

On day 14 post-infection, we reported the effect of ACE2 on the levels of anti-SARS-CoV-2 IgG and IgM antibodies, finding that they were higher in the more severe cases compared to the moderate COVID-19 group. Data from previous studies have shown that most adults with SARS-CoV-2 infection develop virus-specific IgM and IgG antibodies, peaking between days 14 and 20 and then declining [38]. This data, combined with the overall higher cytokine levels in severe COVID-19 patients, showcase a more immense immune response to the infection aiming to irradicate the virus, which, in some cases, can lead to uncontrolled release of cytokines, causing cytokine storm, which can be fatal.

### 4.2. Angiotensin II Serum Levels

Monitoring serum Ang II levels is important because, on one hand, Ang II has a major role in the function of renal and cardiovascular physiology, and, on the other hand, with its role in systemic inflammation, tissue damage, autoimmunity, oxidative stress and aging [39].

Results from the measurement of Ang II serum levels showed similar values in patients and healthy controls, while statistically significant higher levels were reported in the group of vaccinees. These results are consistent with those previously reported by Ozkan and colleagues [40], who found significantly lower serum angiotensin II levels in patients with COVID-19 associated with lung injury [40].

The high values of Ang-II after vaccination indicate that ACE2 is not an exclusive angiotensinase in nature. Other angiotensin convertases (prolyl carboxypeptidase (PRCP) and prolyl oligopeptidase (POP) [41]) are involved in the processing of Ang II to Ang1,7 and may show a countervailing effect on the deleterious interaction between the Spike protein (as in SARS-CoV-2, as well as vaccine-induced) and the ACE2 receptor [42,43].

After binding to the virus and to the Spike protein produced after the mRNA vaccine, ACE2 levels are downregulated, leading to dysregulation in RAAS, an increase in Ang II uptake and activity and an additional decrease of Ang1,7 formation, which exerts the protective effects of ACE2 [44].

Accumulated evidence in this field shows that specific conditions are associated with ACE2 deficiency and dysregulation of the RAAS [45] (extremely advanced age [46], male gender [47], the presence of diabetes [48], lung derivation [49] and history of cardiovascular events [50]), with risk factors for an increased severity of what is reported in COVID-19 [20,21,22]. In our study, we registered a positive influence of Ang-II on the expression of cytokines. We found that increased levels of Ang-II induced increased expression of the cytokines: IL-6, IL-10, IL-33, IL-28A and CD40L in the severe COVID-19 group. However, the absolute value of T cells, their subpopulations (Th and Tc), B lymphocytes and NK cells were decreased in patients with severe COVID-19 compared to milder cases of COVID-19. These results are likely due to decreased proliferation of circulating CD4+ and CD8+ T cells in patients with severe COVID-19. Some authors found that T cells and NK cells showed an exhaustion phenotype with expression of higher levels of exhaustion markers, including programmed cell death protein-1 (PD-1) [51].

Reduced T cell proliferation was also reported by other authors in patients with prolonged hospitalization (>20 days) who tested negative for the virus by PCR at the time of lymphocyte collection [52].

The results from the study of Almutlaq M. (2022) on PBMC’s and purified CD3+ T cells infected with SARS-CoV-2 also showed reduced proliferation [52]. Other authors have reported opposite effects of angiotensin II on T cells, suggesting that they are due to its proliferative effects [53]. These authors found that angiotensin II favors the vascular accumulation of T cells, which are specific endothelial CCR5 + CD4+ T cells and memory/activated CD44high, having the phenotype of effector T cells. These cells have a low activation threshold, produce TNFα and IFNγ and may contribute to vascular dysfunction and hypertension, which is recorded both during the COVID-19 disease and directly after vaccination [52]. TNF alpha, in turn, activates the NF-κB pathway, which leads to the activation of proinflammatory genes whose products are cytokines that activate immune cells excessively for a long time, which may be one of the reasons for their exhaustion and suppressed function [54].

### 4.3. Cathepsin L Serum Levels

To determine the involvement of cathepsins, in the process of developing the infection and after vaccination, we chose to detect the serum levels of cathepsin L. Already at the very beginning of the pandemic, it was found that the virus-ACE2 complex is internalized by clathrin-mediated endocytosis into endolysosomes, where S2′ cleavage by cathepsins, which require an acidic environment for their activity, takes place [55].

Analyzing serum CTSL levels, we found that the vaccinated group had the highest values compared to the other groups, and in some patients with moderate COVID-19, CTSL levels were lower compared to severe COVID-19. Similar results were also reported by Zhao and colleagues, who found that circulating levels of CTSL were increased after SARS-CoV-2 infection and positively correlated with the course and severity of the disease [56]. In laboratory experiments, infection with SARS-CoV-2 pseudovirus was found to increase the expression of CTSL in human cells in vitro and ACE2 in transgenic mice in vivo, demonstrating that overexpression of CTSL enhances viral infection in cells [56].

Furthermore, one of the key functions of CTSL is its involvement in the processing and presentation of antigens by antigen-presenting cells (macrophages, dendritic cells, B cells) [57]. In this regard, a potential explanation for the high levels of CTSL in vaccinated individuals is precisely the relationship between antigen-presenting cells and T and B lymphocytes aimed at initiating a sustained immune response [56]. This process is not short in time although the initial presentation and activation of T cells typically occur within the first few days to a week after vaccination. However, the process does not stop there. Once T cells are activated, they can continue to interact with APCs over an extended period. This prolonged interaction allows for the development of memory T cells, which are a critical component of long-term immunity, and mRNA vaccines are designed to induce cellular memory. Therefore, antigen presentation by the virus continues long after vaccination, which can result in higher levels even 14 days after a second dose of the vaccine.

Measuring the impact of CTSL on the immunoglobulin levels, cytokines and B and T cells, we again observed a positive impact and dramatically increased levels of some cytokines in the subgroup with severe COVID-19.

These results are likely due to the high activity of CTSL, which correlates with markers of inflammation, suggesting that the release of activated CTSL into the circulation occurs in association with ongoing local or systemic inflammation. CTSL is expressed in all tissues and cell types because the main function of cysteine cathepsins is the proteolysis of protein antigens produced by pathogen endocytosis [58].

CTSL has a major role in the stimulation and activation of the body’s immune response by activating M1 macrophages (Hu et al., 2020), which mainly contribute to inflammation and tissue damage during chronic inflammatory processes, and which have the highest expression of cathepsins [59]. Our results support a hypothesis that CTSL may also be involved in triggering the cytokine storm [59].

However, further studies are still needed to establish the role of increased amounts of CTSL in inflammatory cells in inflammatory conditions to support this hypothesis.

### 4.4. Tumor Necrosis Factor Alpha Serum Levels

According to summary statistics by WHO among patients with COVID-19, about 19% had elevated serum levels of proinflammatory cytokines and various immune cells [60]. The dramatic increase in cytokine levels in critically ill patients with COVID-19 is called cytokine release syndrome (CRS). Data obtained from patients with COVID-19 also indicate that severe cases may be characterized by CRS-induced irreversible progression to acute respiratory distress syndrome (ARDS) [60].

In this regard, we compared the serum levels of the main cytokine TNFα in the studied groups.

The measured serum levels of TNFα were almost three times higher in vaccinated compared to healthy subjects. We also reported increased levels in patients with moderate and severe COVID-19. Correlation analysis also showed a positive influence of TNFα on the studied cytokines: IL-10, IL-33, IL-28A and CD40L. 

Reduced levels of T cells and their function in severe COVID-19 may be due to lower levels of IL-6, which is secreted by T cells, macrophages, endothelial cells, fibroblasts and monocytes [61]. The targets of IL-6 are B-cells, T-cells, basophils, eosinophils and neutrophils. The effect of IL-6 on B-cells is expressed in the stimulation of B-cell differentiation, as well as the production of total IgM, IgG and IgA antibodies. In our case, immunoglobulin levels fluctuated between individual subgroups, and a direct relationship could not be established with IL-6. In the case of patients with severe COVID-19, the high levels of T cells may be stimulated by cytokines other than IL-6. TNFα is also a key proinflammatory marker, and its elevated levels can condition T cell numbers. Moreover, high levels of T lymphocytes do not guarantee their optimal functionality, due to phenomena such as cellular exhaustion.

Previous studies have found that very high levels of TNFα portend a poor prognosis in cases of SARS-CoV and MERS [62] and that the inhibition of NF-κB ameliorates pulmonary symptoms in SARS-CoV-infected mice [62]. However, opposite results have been reported by researchers, according to which non-specific blocking of NF-κB can simultaneously disrupt its protective functions in cellular homeostasis, as well as cause a general suppression of innate immunity [63]. We could not qualify high TNFα levels as disease outcome predictors in this study.

When determining the effect of TNFα on serum cytokine levels, we found positive correlation with NK cells, IL-28A and CD40L.

Natural killer cells (NK cells) are a type of immune cell that can recognize and eliminate virus-infected cells and cancer cells. The correlation we observed between higher levels of TNFα and increased NK-cell numbers indicates that there might be a positive feedback loop in play.

Elevated TNFα levels might stimulate the activation and expansion of NK cells, enhancing their cytotoxic and antiviral functions [64]. NK cells can respond quickly to infections and contribute to the early defense against viral pathogens. This correlation could suggest a coordinated immune response to combat the infection, where TNFα acts as a trigger for NK cell activation.

The correlation between TNFα and IL-28a levels might indicate a relationship between proinflammatory cytokines and antiviral responses. IL-28a is a type III interferon that is part of the antiviral immune response. The correlation could suggest a potential regulatory mechanism where proinflammatory signals like TNFα contribute to the production of antiviral cytokines like IL-28a, possibly enhancing the immune response against the virus. We found somewhat similar findings in a study, where the researchers reported an elevation in mRNA levels of both IL-28 and TNF in dendritic cells [65]. However, in the context of COVID-19, we did not find similar results in the literature, stating an evident correlation between serum levels of those two biomarkers.

CD40L (CD154) is a co-stimulatory molecule expressed on activated T cells, which plays a critical role in B cell activation and antibody production. The correlation between TNFα and CD40L levels could imply a connection between the proinflammatory environment and immune cell activation. The correlation between these two markers has been reported before by other authors in different diseases (Crohn’s disease, heart failure, major injuries) [66,67,68]. It is possible that the elevated TNFα levels contribute to the upregulation of CD40L expression, or vice versa, promoting B cell responses and antibody production. 

The NF-κB pathway is a major signaling pathway that regulates the expression of genes involved in inflammation, immune responses and cell survival [69]. TNFα is a well-known activator of NF-κB [70]. When TNFα binds to its receptor on cell surfaces, it triggers a series of intracellular events that lead to the activation of NF-κB. This, in turn, induces the transcription of various genes, including those encoding inflammatory cytokines, adhesion molecules and immune cell co-stimulatory molecules [70].

The observed correlation between TNFα and CD40L levels could be indicative of a potential feedback loop. Higher levels of TNFα could activate the NF-κB pathway in various immune cells, including antigen-presenting cells. This pathway activation might then contribute to the upregulation of CD40L expression on activated T cells. The interaction between CD40L and CD40 on antigen-presenting cells could further potentiate NF-κB activation and immune cell activation, creating a positive feedback loop, resulting in higher cell numbers, since NF-κB activation causes apoptosis evasion, and mounting a stronger inflammatory response.

Overall, these correlations provide insights into the complex immune response dynamics during COVID-19 and vaccination. The interactions between TNFα and various immune markers highlight the intricate balance between proinflammatory signals, antiviral responses, immune cell activation and the immune system’s attempt to control the infection. Further research is needed to elucidate the underlying mechanisms and the implications of these correlations for disease progression, immune memory development and potential therapeutic interventions.

### 4.5. mRNA Vaccines

Analyzing the relationship between vaccination and the high serum concentrations of the markers studied by us, we can assume that they are due to the characteristics of the vaccine itself. Research on the effectiveness of different vaccines shows that vaccination activates the body’s immune response [71]. Previous studies have reported an increase in the immune response as well as systemic side effects in individuals with increased levels of IFN-γ, TNF-α, IL-2, IL-5 and IL-10 after vaccination [72]. 

Studies on the level of circulating ACE2 and other markers have shown generally low amounts in healthy people [73], which is expected. In our case, in addition to healthy individuals, we also reported low values in patients with COVID-19, which was probably due to the fact that the samples were collected on the 14th day after admission to the hospital. Among vaccinated individuals, ACE2, Ang II, CTSL and TNFα had very high serum levels. To investigate the reasons for these results, we checked the post-vaccination reports and found that Robles et al. [74], when experimenting on mice, discovered that after vaccination with mRNA vaccine, the spike protein caused endothelial cells (ECs) dysfunction [70]. Studies have demonstrated that the spike protein of SARS-CoV-2 independently activates the inflammatory phenotype of ECs and induces the nuclear translocation of NF-κB and subsequent expression of leukocyte adhesion molecules (VCAM-1 and ICAM-1), coagulation factors, proinflammatory cytokines (TNFα IL-1β and IL-6) and ACE2 [72,73,74]. These results are consistent with our measured high serum ACE2, AngII, CTSL and TNFα values.

The main vaccines against SARS-CoV-2 use a molecule called messenger RNA (mRNA). These vaccines use this mRNA to direct cells toward producing copies of the the spike protein, which is usually found on the outside of the coronavirus [43,72,73].

However, some concerns have been raised about the safety of SARS-CoV-2 vaccines, mostly based on occurring events such as thromboembolism [75], myocarditis and myopericarditis [76] and elevated BP [76] after vaccination.

From our group of vaccinated individuals, only one woman reported the occurrence of complications (weakness, numbness, headache, dizziness, dyspnea, fatigue, muscle spasms, joint pain), which was also reported by other authors [77]. This is due to the specificity of this type of vaccine, which uses the viral spike protein, the synthesis of which is induced by the mRNA. Its binding to ACE2 receptors triggers the same process that occurs after infection with the virus. The result of this process is a decrease in the activity of ACE2 on the cell surfaces and therefore a deficiency of angiotensin 1-7 and an excess of angiotensin II [78]. This excess of angiotensin II, unbalanced by angiotensin 1-7, is responsible for the increase in blood pressure, for thrombotic and inflammatory phenomena (although they are within 1/100,000), in greater probability after secondary vaccination, a pathological phenomenon characteristic of SARS-CoV-2 infection [79].

Although there is a low risk of complications after vaccination, the advantage of vaccination against COVID-19 over non-vaccination is an indisputable fact worldwide [75]. The main advantage of vaccines is related to the prevented hospitalizations and serious complications of COVID-19, as well as the long-term effects of the so-called ‘long COVID-19’ on human health. It is likely that in future developments, these negative effects will be taken into account by editing the mRNA molecule of the vaccines, and these shortcomings will be removed, improving their effectiveness.

Our study has some limitations. First, this study included a relatively small number of patients. Our results deserve to be confirmed in larger studies. Second, we had access to a limited number of samples of severe COVID-19, only from patients in the intensive care unit.

The number of vaccinated and healthy persons is also a small number because, at that time, there was a fear of vaccines. Gender segregation further reduced the number of participants per group. Therefore, determining potential relationships by sex, age and clinical parameters will require further research.

## 5. Conclusions

The intricate interplay observed among ACE2, CTSL, AngII, TNFα, serum cytokines and antibody responses add layers of complexity to our understanding of the immune responses triggered by SARS-CoV-2 infection and mRNA vaccination. These findings unveil a dynamic and multifaceted immune landscape that not only responds to viral intrusion but also reacts to the introduction of mRNA vaccines.

One of the central implications of these findings lies in the potential key role of ACE2 in modulating immune responses during severe COVID-19 cases. The positive correlations between ACE2 and proinflammatory cytokines IL-33 and CD40L point to ACE2 as a potential driver of the heightened inflammatory response observed in severe cases. Further research could explore the underlying mechanisms behind this relationship, potentially leading to the identification of novel therapeutic targets for mitigating severe COVID-19 outcomes.

Similarly, the connection between AngII and proinflammatory markers such as CD40L and IL-33 suggests a potential role for the renin-angiotensin system in influencing immune responses. Investigating the crosstalk between AngII and these markers may yield insights into the mechanisms driving immune dysregulation in severe cases. Strategies aimed at modulating this axis could hold promise in managing COVID-19 severity.

The positive associations found between CTSL and IgA and IL-28A levels point toward the intricate role of proteases in shaping antibody responses and antiviral cytokine signaling. Understanding how CTSL influences these immune components may offer avenues for optimizing vaccine-induced immunity and enhancing our capacity to combat SARS-CoV-2 and other viral infections.

In summary, the findings presented in this study unveil a complex web of interactions within the immune system in response to SARS-CoV-2 infection and vaccination. These discoveries not only enhance our comprehension of COVID-19 pathogenesis but also provide a roadmap for future research and therapeutic development. The identified correlations and associations serve as signposts, guiding us toward a more nuanced understanding of immune responses in the context of COVID-19. The ongoing exploration of these intricate connections promises to inform strategies for managing COVID-19, optimizing vaccine responses and advancing our capacity to tackle viral infections effectively. Continued investigation into these multifaceted immune dynamics will be paramount in the quest to control and ultimately conquer the COVID-19 pandemic.

## Figures and Tables

**Table 1:** Mean values of the serum markers in different groups based on disease severity. Table contains mean and *p* values from the Mann–Whitney test; * *p* < 0.05.

**▪**	ACE2 (pg/mL)			AngII (pg/mL)			CTSL (pg/mL)			TNFα (pg/mL)		
**▪**	Moderete COVID-19	Severe COVID-19	*p*	Moderete COVID-19	Severe COVID-19	*p*	Moderete COVID-19	Severe COVID-19	*p*	Moderete COVID-19	Severe COVID-19	*p*
**▪n**	21	6		17	9		16	11		25	2	
**▪Marker mean**	326.57 ± 854.84	1428.75 ± 1386.70		155.52 ± 141.60	275.52 ± 191.28		1286.54 ± 1084.15	2080.73 ± 1284.15		253.54 ± 283.66	1175.24 ± 232.10	
**▪IgG (g/L)**	9.27 ± 5.28	7.96 ± 2.05	0.798	9.65 ± 5.82	7.60 ± 1.60	0.312	8.08 ± 2.07	10.28 ± 7.01	0.645	9.08 ± 4.92	7.66 ± 1.87	0.627
**▪IgA (g/L)**	2.02 ± 0.74	2.35 ± 1.51	0.932	2.01 ± 0.69	2.40 ± 1.27	0.560	1.68 ± 0.52	2.70 ± 1.09	0.005 *	1.94 ± 0.71	4.08 ± 1.47	0.023 *
**▪IgM (g/L)**	1.55 ± 0.57	0.97 ± 0.51	0.042 *	1.49 ± 0.45	1.28 ± 0.84	0.133	1.49 ± 0.40	1.31 ± 0.82	0.162	1.49 ± 0.56	0.57 ± 0.26	0.011 *
**▪IL-6 (pg/mL)**	27.94 ± 43.03	17.51 ± 20.31	0.974	23.29 ± 44.15	29.05 ± 30.64	0.294	29.73 ± 45.51	19.18 ± 26.80	0.880	27.22 ± 39.69	4.59 ± 2.67	0.406
**▪IL-10 (pg/mL)**	319.75 ± 540.46	844.39 ± 1088.23	0.744	346.19 ± 584.72	681.27 ± 967.85	0.754	360.25 ± 621.27	550.69 ± 843.06	0.799	290.89 ± 495.84	2240.00 ± 270.11	0.025 *
**▪IL-33 (ng/L)**	17.65 ± 7.46	52.80 ± 47.40	0.009 *	17.80 ± 7.95	43.29 ± 43.79	0.175	18.56 ± 7.81	35.57 ± 39.12	0.610	18.46 ± 7.04	113.33 ± 15.32	0.006 *
**▪IL-28A (ng/mL)**	381.52 ± 280.46	981.17 ± 973.49	0.295	352.59 ± 236.27	904.51 ± 868.98	0.157	296.72 ± 263.74	824.24 ± 717.82	0.013 *	381.23 ± 281.84	2184.00 ± 255.97	0.006 *
**▪CD40L (ng/mL)**	1.98 ± 1.32	15.81 ± 26.49	0.006 *	1.75 ± 0.99	12.56 ± 23.22	0.023 *	2.57 ± 1.45	8.71 ± 20.39	0.357	2.16 ± 1.35	41.26 ± 39.52	0.006 *
**▪T-cell**	1074.99 ± 767.05	1472.19 ± 988.67	0.550	1291.79 ± 877.34	978.42 ± 725.86	0.339	986.13 ± 612.55	1420.90 ± 1026.18	0.512	1153.94 ± 823.77	1279.71 ± 1036.87	0.963
**▪T-cytotoxic cells**	421.82 ± 359.34	559.16 ± 371.08	0.376	513.14 ± 389.90	361.44 ± 304.76	0.396	332.50 ± 200.06	626.67 ± 468.46	0.195	436.85 ± 351.03	645.87 ± 566.40	0.570
**▪T-helper cells**	611.03 ± 488.42	853.01 ± 626.41	0.512	731.42 ± 562.69	577.66 ± 457.61	0.426	617.46 ± 404.94	733.67 ± 667.72	0.865	671.56 ± 532.39	580.30 ± 433.92	0.889
**▪NK-cells**	169.88 ± 127.41	359.28 ± 202.27	0.049 *	223.13 ± 156.28	206.00 ± 188.73	0.711	205.56 ± 148.36	221.29 ± 191.02	0.981	192.68 ± 148.79	453.07 ± 199.42	0.091
**B-cells**	216.25 ± 138.89	263.35 ± 149.09	0.589	233.73 ± 132.56	219.12 ± 166.66	0.491	201.36 ± 127.20	263.60 ± 154.72	0.342	233.92 ± 142.38	136.67 ± 57.03	0.410

**Table 2:** Correlation values between biomarkers levels (ACE2, AngII, CTSL, TNFα) and cytokines, T cells and B cells in serum. Table includes Spearman’s rho Correlation Coefficient; * Correlation is significant at the 0.05 level (2-tailed); ** Correlation is significant at the 0.01 level (2-tailed).

	ACE2	AngII	CTSL	TNF-α
IgG (g/L)	0.061	−0.177	0.035	−0.038
IgA (g/L)	0.008	0.102	0.437 *	0.258
IgM (g/L)	−0.273	−0.129	−0.273	−0.180
IL-6 (pg/mL)	−0.047	−0.074	−0.037	−0.346
IL-10 (pg/mL)	0.094	0.196	0.095	−0.168
IL-33 (ng/L)	0.539 **	0.416 *	0.182	0.239
IL-28A (ng/mL)	0.275	0.096	0.592 **	0.491 *
CD40L (ng/mL)	0.520 **	0.504 *	0.045	0.458 *
T-cells (×10^9^/L)	0.006	−0.382	−0.122	0.156
T-cytotoxic cells (×10^9^/L)	0.038	−0.336	−0.014	0.049
T-helper cells (×10^9^/L)	0.021	−0.313	−0.252	0.178
NK-cells (×10^9^/L)	0.261	−0.011	−0.176	0.190
B-cells (×10^9^/L)	0.025	−0.239	−0.092	0.094

## Data Availability

Data are contained within the article and Supplementary Materials.

## Supplementary Materials

The following supporting information can be downloaded at: https://www.mdpi.com/article/10.3390/biomedicines11123160/s1, Table S1: Characteristics of COVID-19 patients.
