# Supplementary material for: The Serum ACE2, CTSL, AngII, and TNFα Levels after COVID-19 and mRNA Vaccines: The Molecular Basis
Source: Biomedicines. 2023 Nov 27;11(12):3160. doi: 10.3390/biomedicines11123160 (PMC10741205; doi:10.3390/biomedicines11123160)
Supplement: Supplementary file 1 [file biomedicines-11-03160-s001.zip › biomedicines-2699637-supplementary.pdf]

Table S1. Characteristics of COVID-19 patients

Demographic characteristics					
COVID-19		Vaccinees		Healthy	
N	Median age	N	Median age	N	Median age
63	72 (23-87)	49	45 (27-60)	37	54 (35-69)

Laboratory results of COVID-19 patients						
	Moderate COVID-19			Severe COVID-19		
	N	Mean	SD	N	Mean	SD
Hemoglobin (HGB)						
< [120 -160]	2	112,00	1,45			
[120 -160]				25	135,93	10,421
Red blood cells (RBCs)						
[3,9 -5,3]	21	4,4304	0,34123			
> [3,9 -5,3]				6	5,4567	0,16525
White blood cells (WBCs)						
< [3,5 -10,5]	4	3,4300	0,06928			
[3,5 -10,5]	17	6,9640	2,44546			
> [3,5 -10,5]				6	15,9267	1,12037
Platelets (PLT)						
< [140 -400]	6	102,00	26,713			
[140 -400]				21	213,58	64,188
Neutrophil granulocytes						
(Sg)	2	55,700	0,076			
[42 -70]				25	84,321	7,1403
>[42 -70]						
Monocytes (MONO)						
< [5,8 -11,8]	21	3,550	0,9873			
[5,8 -11,8]				6	7,933	1,7072
Lymphocytes (LYMPH)						
< [1,3 -3,9]	25	0,6850	0,25891			
[1,3 -3,9]				2	2,4200	0,00000
Lymphocytes (LYMPH)						
< [22 -48]	25	10,593	5,5363			
[22 -48]				2	27,500	0,0000
Westergren (ESR)						
[2 -30]	8	18,625	6,1339			
>[2 -30]				19	64,318	12,5333
Fibrinogen						
(Fbg)	4	2,7250	0,53694			
[2,0 -4,5]				23	6,0935	1,00046
> [2,0 -4,5]						
D-Dimer						

[0,0 -0,5]<br>> [0,0 -0,5]	2	0,4400	0,0012	25	3,0961	2,87166
Glucose (GLUC)<br>[2,8 -6,1]<br>> [2,8 -6,1]	2	5,900	0,0032	25	9,054	3,1961
C-reactive protein (CRP)<br>[0 -10]<br>> [0 -10]	2	5,000	0,057	25	82,150	34,4617
AcAT (AST)<br>[0 -36]<br>> [0 -36]	8	28,89	1,900	19	79,95	31,313
ALAT, SGPT<br>[0 -35]<br>> [0 -35]	10	23,80	4,022	17	1153,21	1255,103
Lactate dehydrogenase (LDH)<br>< [230 -460]<br>[230 -460]<br>> [230 -460]	11<br>2	75,91<br>434,00	56,444<br>8,5	14	1120,18	430,802

Data for experimental variables

	Mean	95.0% Lower CL for Mean	95.0% Upper CL for Mean	Standard Deviation	Minimum	Maximum	Valid N
IgG g/L	8.978	7.097	10.859	4.754	4.676	30.543	27
IgA g/L	2.097	1.727	2.467	0.936	0.783	5.120	27
IgM g/L	1.421	1.184	1.657	0.597	0.389	3.181	27
IL-6 (pg/mL)	25.335	9.091	41.578	38.467	0.500	176.000	24
IL-10 (pg/mL)	440.824	152.474	729.174	713.899	53.650	2553.000	26
IL-33 (ng/L)	25.760	14.925	36.596	26.828	0.532	124.170	26
IL-28A (ng/mL)	519.904	292.959	746.848	561.871	33.710	2365.000	26
CD40L (ng/mL)	5.170	-0.205	10.544	13.307	0.655	69.210	26
T- cell	1163.257	839.726	1486.787	817.850	163.509	2833.566	27
T- cytotoxic cells	452.342	310.154	594.529	359.435	46.863	1344.144	27
T- helper cells	664.803	459.451	870.155	519.108	58.030	2031.965	27
NK- cells	211.972	147.218	276.725	163.689	16.598	594.083	27
B-cells	226.720	171.462	281.979	139.687	39.789	500.463	27

Used COVID-19 vaccines

N	Age	COVID-19 Vaccines	Vaccine Type/Platform	Developer of the Vaccines	Dose
27	38.0 (27.0–40.0)	Pfizer-BNT162b2	Nucleoside-modified mRNA	BioNTech SE, Pfizer Inc.	Two doses given three weeks apart
22	54.0 (47.0–60.0)	Moderna vaccine (mRNA-1273)	Nucleoside-modified mRNA	United States National Institute of Allergy and Infectious Diseases, Moderna Inc.	Two doses given four weeks apart
